# Gene response in rice plants treated with continuous fog influenced by pH, was similar to that treated with biotic stress

**DOI:** 10.1186/s12284-014-0010-9

**Published:** 2014-06-11

**Authors:** Kouji Satoh, Shoko Saji, Shoko Ito, Hideyuki Shimizu, Hikaru Saji, Shoshi Kikuchi

**Affiliations:** 1 Plant Genome Research Unit, Agrogenomics Research Center, National Institute of Agrobiological Sciences (NIAS), Tsukuba 305-8602, Ibaraki, Japan; 2 Center for Environmental Biology and Ecosystem Studies, National Institute for Environmental Studies (NIES), Tsukuba 305-8506, Ibaraki, Japan; 3 Center for Regional Environmental Research, NIES, Tsukuba 305-8506, Ibaraki, Japan; 4 Present address: NARO Hokkaido Agricultural research Center, National Agricultural research Organization, Sapporo 062-8555, Hokkaido, Japan

**Keywords:** Rice, Acid fog, Oxidative stress, Transcriptome, Comparative analysis

## Abstract

**Background:**

Throughout Asia, including Japan, rice plants are cultivated in a wide range of areas from lowlands to highlands and are frequently exposed to fog, including acid fog. Some physiological studies have shown that acid fog can be a stress factor for plants. We analyzed the gene expression profiles of rice plants treated with artificially prepared simulated acid fog (SiAF) or simulated neutral fog (SiNF) for 1 or 7 days.

**Results:**

Microarray analysis results suggested that both the SiAF and the SiNF treatments induced the expression of genes involved in the defense and stress responses in rice plants. Induction of such genes was detected in plants treated with SiAF for 1 day, and the number of induced genes increased in plants treated with SiAF for 7 days. The genes for defense and stress responses were also induced by SiNF for 7 days, although they were not induced by SiNF for 1 day. The gene expression profiles of the SiAF-treated and the SiNF-treated plants were compared to those of plants treated with other stress factors. The comparison revealed that both SiAF and SiNF treatments have similar effects to biotic stresses and ozone stress. The genes encoding NADPH oxidase and germin, which function in apoplasts, were also induced by SiAF, SiNF and biotic stresses.

**Conclusions:**

These findings suggest that both the SiAF and the SiNF treatments may result in oxidative stress through the apoplastic production of reactive oxygen species.

## Background

Sulfur dioxide (SO_2_) and nitrogen oxides (NOx) emitted into the atmosphere react with moisture to form acidic solutions, resulting in acid rain or fog. Investigations of the effects of acid rain or fog on plants have shown that these factors have negative effects on plant growth, including lesion formation, chlorosis, and reduction of biomass (Ferenbaugh [1976]; Igawa and Okochi [2009]), the extent of which depends on the pH and the frequency of the treatment (Evans et al. [1977]). In *Arabidopsis*, acid rain treatment results in symptom development and the induction of genes in the signaling pathway mediated by salicylic acid (SA). Moreover, SA-mediated gene responses were shown to be induced by acid rain containing sulfate but not by acid rain containing nitrate (Lee et al. [2006]).

Acid rain treatment is also known to generate reactive oxygen species (ROS) in plants (Gabara et al. [2003]; Velikova et al. [2000]; Wyrwicka and Sklodowska [2006]), and ROS are associated with the disturbance of organelle structures (Gabara et al. [2003]; Torres [2010]). Gaseous SO_2_ treatment also induces ROS generation in plants, and ROS accumulate in the apoplast (Li et al. [2007]). The type and timing of antioxidant gene induction is dependent on the plant species (Velikova et al. [2000]; Wyrwicka and Sklodowska [2006]). Similarly, the severity of symptoms induced by acid rain treatment is also dependent on the plant species (Haines et al. [1980]; Wang et al. [2008]).

Rice is a major crop cultivated in both lowland and mountainous (highland) areas in Asia, including Japan. Fog events occur more frequently in highland areas because the temperature differences between day and night and between different sites are greater than those in lowland areas. Rice plants cultivated in highlands are therefore frequently exposed not only to fog but also to acid fog. Generally, the pH of acid fog is lower than that of acid rain. In Japan, acid fog has been detected in highlands (Igawa et al. [2001]) and has been suggested to be associated with the growth injury to rice that is sometimes observed in highland areas in Nagasaki, Kyushu.

Rice is known to show resistance to acid rain (Wang et al. [2008]), but the mechanism of this resistance has not been investigated. Moreover, the gene responses of rice plants to acid fog treatment have not been investigated. In the present study, therefore, we have analyzed the gene responses in rice plants treated not only with an acid fog but also with a neutral fog, and we have conducted a comparative analysis of the gene responses between fog treatment and other stress treatments to investigate possible common regulatory mechanisms between fog treatment and other abiotic and biotic stresses.

## Results

### Gene responses induced by fog treatments

Visual symptoms were not observed in either the simulated acid fog (SIAF)-treated or the simulated neutral fog (SiNF)-treated rice plants at the end of the 7-day fog treatments. Symptoms such as chlorosis were, however, observed on leaves treated with SiAF for 14 days (Ito et al. [2011]). To analyze whether rice plants responded to either the SiAF or the SiNF treatment at a biochemical level during the 7-day fog treatments, we analyzed the gene responses in both the SiAF- and the SiNF-treated plants by using a 60-mer oligonucleotide microarray.

**Figure 1:**
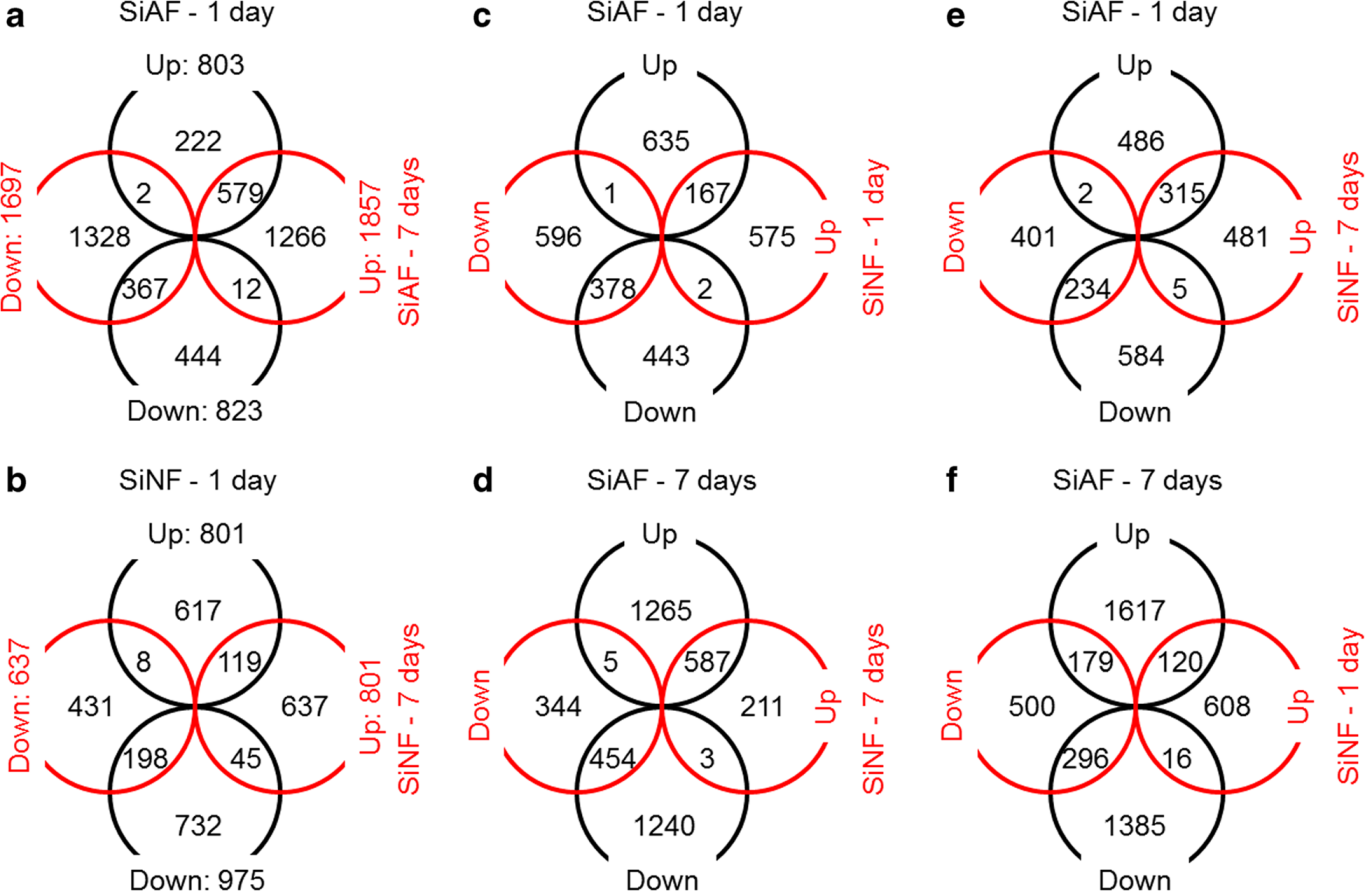
**Similarity of gene responses across different fog treatments.** The numbers of up- and down-regulated genes by either one or both of SiAF-1day and SiAF-7day treatments are shown in panel **a**, and those in other combinations of different fog treatments are similarly shown in panels **b** to **f**.

The expression of 22,328 genes was detected in at least one condition by this microarray system (Additional file 1: Table S1). The number of significantly differential expressed genes (DEGs) in the SiAF-treated rice plants was dependent on the length of treatment: 1,626 and 3,554 genes in plants treated for 1 and 7 days, respectively (Figure 1a). The frequency of commonly activated DEGs between the 1- and 7-day SiAF-treated plants was higher than that of commonly suppressed DEGs (Figure 1a). The number of DEGs was similar, at 1,719 and 1,438 genes, respectively, between rice plants that underwent SiNF treatment for 1 and 7 days (Figure 1b). The frequency of common DEGs between plants treated with SiNF for 1 and 7 days was lower than that between plants treated with SiAF for 1 and 7 days (Figure 1a and b). Some of the DEGs detected by the microarray analysis were also analyzed by RT-PCR, and the gene responses detected by the microarray analysis were confirmed by the RT-PCR results (Additional file 2: Figure S1).

DEGs were also compared between the SiAF- and SiNF-treated plants (Figure 1c-f), and more than 70% of the DEGs in plants treated with SiNF for 7 days (1,039 of 1,438) responded similarly in plants treated with SiAF for 7 days (Figure 1d), although only approximately 30% of the DEGs in plants treated with SiNF for 1 day (545 of 1,719) responded to the 1-day SiAF treatment (Figure 1c). Moreover, 195 of the DEGs in plants treated with SiNF for 1 day demonstrated the opposite response (upregulated in one and downregulated in the other) compared with plants exposed to the 7-day SiAF treatment (Figure 1f). Therefore, the effect of the 1-day SiNF treatment on gene expression appears to substantially differ from those of other fog treatments.

### ROS generation and antioxidant systems

**Figure 2:**
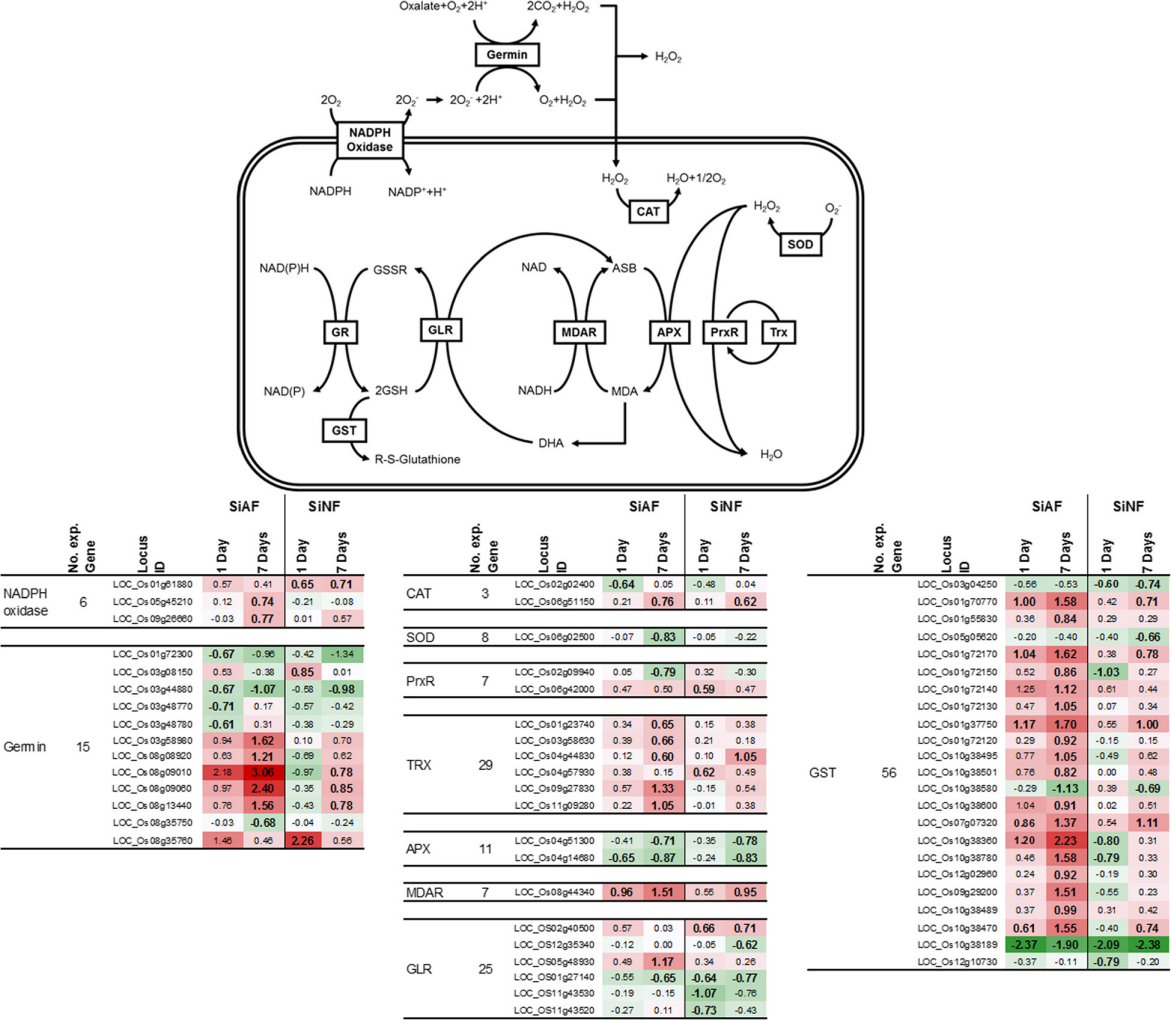
**Generation and reduction of reactive oxygen species.** SOD: Superoxide dismutase; APX: Ascorbate peroxidase; MDAR: MDA reductase; DHAR: DHA reductase; GLR: Glutaredoxin; GR: Glutathion reductase; CAT: Catalase; PrxR: Peroxiredoxin; Trx: Thioredoxin; NADPH: Nicotinamide adenine dinucleotide phosphate; ASB: ascorbate; MDA: Monodehydroascorbate; NAD, NADH: Nicotinamide adenine dinucleotide; GSSR: Oxidized glutathione; GSH: Reduced glutathione; DHA: Dehydroascrobate. This pathway is referenced by Miller et al. ([2010]) . Red and green colors indicate up- and down-regulation of gene expression, respectively. The number represents the log _2_ ratio (signal intensity of the treated sample/signal intensity of the control sample).

ROS production and scavenging occur at different sites in cells, including the apoplast, cytosol, and various organelles (Torres [2010]; Gill and Tuteja [2010]; Miller et al. [2010]). In the apoplast, O_2_^−^ is produced by NADPH oxidases located on the plasma membrane (Gill and Tuteja [2010]; Miller et al. [2010]; Torres [2010]). Two genes encoding NADPH oxidases (LOC_Os05g45210, LOC_Os09g26660) were activated by the 7-day SiAF treatment (Figure 2, Additional file 1: Table S1). Another gene encoding an NADPH oxidase (LOC_Os01g61880) was also induced by the 7-day SiNF treatment (Figure 2). The germin protein family is a pathogenesis-related (PR) protein family that functions at the apoplast (Manosalva et al. [2009]; van Loon et al. [2006]) and reduces O_2_^−^ to H_2_O_2_ (Banerjee and Maiti [2010]; Gill and Tuteja [2010]). The expression of certain germin-encoding genes (LOC_Os03g58980, LOC_Os08g08920, LOC_Os08g09010, LOC_Os08g09060, LOC_Os08g13440) was activated in plants treated with SiAF for 7 days (Figure 2). Moreover, the expression of germin-encoding genes was also activated by the 1- and 7-day SiNF treatment; however, the number of induced genes and the degree of induction in the 7-day SiNF-treated plants was less than those in the 7-day SiAF-treated plants.

Electron transport chain systems in the mitochondria, chloroplasts, and peroxisomes are sources of ROS generation, and the reduction of organelle function leads to the overproduction of ROS (Gill and Tuteja [2010]; Miller et al. [2010]; Torres [2010]). However, the microarray data indicate that the expression of genes important for the function of these organelles was not affected by either the SiAF or the SiNF treatment (Table 1). The antioxidant systems in the organelles and cytosol comprise the same family of genes (Gill and Tuteja [2010]; Miller et al. [2010]; Torres [2010]). The SiAF treatment induced many genes encoding glutathione *S*-transferase (GST) and thioredoxin (TRX) (Figure 2), although a few genes encoding other ROS scavengers were also changed by fog treatment (Figure 2). The induction of GST genes was detected in plants treated with SiAF for 1 and 7 days, but the induction of TRX genes was detected only in plants treated with SiAF for 7 days (Figure 2). The genes encoding GST and TRX were also induced by the 7-day SiNF treatment (Figure 2); however, the number of DEGs and the degree of their responses to SiNF treatment were smaller than those of the 1-day SiAF treatment (Figure 2).

**Table 1:** The number of differentially expressed genes in cellular systems

**Category**^**1**^	**No. of genes**^**2**^	**SiAF 1 day**^**3**^	**SiAF 7 days**^**3**^	**SiNF 1 day**^**3**^	**SiNF 7 days**^**3**^
Citrate cycle (TCA cycle)	42	2 / 0	1 / 1	1 / 0	1 / 0
Oxidative phosphorylation	91	3 / 1	3 / 1	1 / 0	2 / 0
Carbon fixation in photosynthetic organisms	62	5 / 0	5 / 3	2 / 1	0 / 5
Photosynthesis	38	0 / 0	1 / 3	0 / 2	0 / 2
Photosynthesis - antenna proteins	9	0 / 1	0 / 2	0 / 0	0 / 0
Peroxisome	49	1 / 1	5 / 2	0 / 1	2 / 0
ET synthesis	3	1 / 0	2 / 0	0 / 0	1 / 0
SA synthesis	21	1 / 1	3 / 2	0 / 1	1 / 1
ABA synthesis	11	1 / 1	1 / 1	2 / 0	0 / 1
AP2-EREBP	77	6 / 4	10 / 5	6 / 4	8 / 3
WRKY	59	11 / 1	21 / 6	1 / 6	5 / 3
PR 2	32	4 / 2	8 / 2	3 / 2	5 / 1
PR 5	22	1 / 1	9 / 2	1 / 5	5 / 0
PR 7	27	1 / 2	6 / 4	2 / 2	2 / 2
PR 10	5	0 / 1	3 / 2	0 / 3	1 / 1
PR 12	6	0 / 2	0 / 2	0 / 3	0 / 2
PR 14	10	0 / 2	0 / 1	0 / 2	0 / 2
Expansin and expansin-like protein	15	0 / 5	1 / 3	0 / 0	1 / 3
Cellulose synthase and cellulose synthase-like protein	26	2 / 0	4 / 1	3 / 0	3 / 0

^1^The gene list of each category is based on KEGG ontology (Kanehisa et al. [2012]), Plant transcription factor database (Zhang et al. [2011]) and MSU OSA1 (Ouyang et al. [2007]).

^2^The number of expressed genes in each category.

^3^The number of differentially activated genes/differentially suppressed genes.

### Defense system

**Figure 3:**
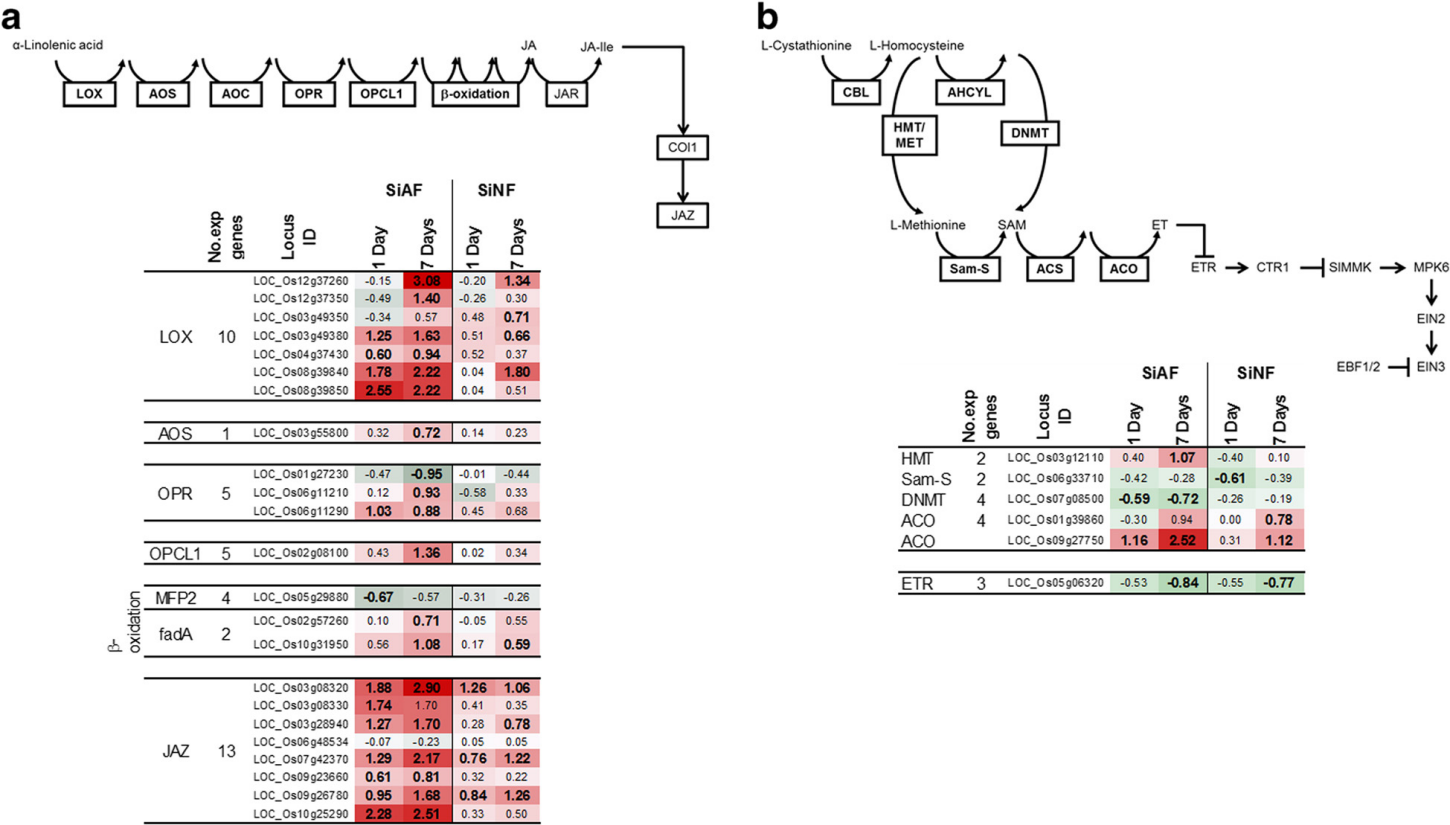
**JA and ET synthesis and signaling pathway. a)** JA synthesis and signaling. LOX: Lipoxygenase; AOS: Allene oxide synthase; AOC: Allene oxide cyclase; OPR: 12-Oxophytodienoic acid reductase; OPCL1: 4-coumarate-CoA ligase 1; MFP2: Glyoxysomal fatty acid beta-oxidation multifunctional protein; fadA: Acetyl-CoA acyltransferase; COI1: 4-Coumarate-CoA ligase1; JAZ: Jasmonate Zim domain. **b)** ET synthesis and signaling. CBL: Cystathionine beta-lyase; HMT: Homocysteine S-methyltransferase; MET: 5-Methyltetrahydropteroyltriglutamate--homocysteine methyltransferase; AHCYL: Adenosylhomocysteinase; DNMT: DNA (cytosine-5-)-methyltransferase; Sam-S: S-adenosylmethionine synthetase; ACS: 1-aminocyclopropane-1-carboxylate synthase; ACO: Aminocyclopropanecarboxylate oxidase; ETR: Ethylene receptor; CTR1: Constitutive triple response1; SIMMK: Salt stress-induced MAPK kinase; EIN2,3: Ethylene-insensitive protein2, 3; EBF1/2: EIN3-binding F-box protein; SAM: S-Adenosyl-L-methionine. The gene list of each pathway is referenced in the KEGG database (Kanehisa et al. [2012]), and Plant transcription factor database (Zhang et al. [2011]). Red and green colors show up- and down-regulation in gene expression, respectively. Numbers represent the log^2^ ratio (signal intensity of the treated sample/signal intensity of the control sample).

The genes involved in defense processes in plants respond to both biotic and abiotic stress conditions, and expression of these genes is controlled by plant hormones such as jasmonic acid (JA), ethylene (ET), SA, and abscisic acid (ABA) (Fujita et al. [2006]; Horváth et al. [2007]). Genes necessary for JA synthesis (Wasternack [2007]) were induced not only by the 1- and 7-day SiAF treatments but also by the 7-day SiNF treatment (Figure 3a). The induction was detected at some reaction steps in JA synthesis; moreover, the gene encoding lipoxygenase (LOX) was highly induced by fog treatment. The expression of JAZ (Jasmonate ZIM domain) genes, which are JA responsive (Ye et al. [2009]), was also activated by both the SiAF and the SiNF treatments. Moreover, the degree of gene induction in plants treated with SiAF for 7 days was higher than that of plants treated with SiNF for 7 days (Figure 3). The genes required for ET synthesis were also induced in plants treated with either SiAF or SiNF for 7-days, and the degree of induction by SiAF treatment was again higher than that by SiNF treatment (Figure 3b). However, the frequency of induced genes in ET synthesis was lower than that in JA synthesis. Moreover, almost all genes involved in ET signaling did not respond to fog treatment. The expression of genes for SA synthesis varied in plants treated with SiAF for 7 days, although SA synthesis itself did not appear to be induced by SiAF treatment (Table 1). Genes for ABA synthesis were not induced by either the SiAF or the SiNF treatment (Table 1).

In hormone-mediated defense systems, transcription factors such as those belonging to the AP2-EREBP and WRKY families control the expression of defense-related genes such as the PR proteins (Pré et al. [2008], Shimono et al. [2007]). The genes encoding the AP2-EREBP proteins were induced not only by the SiAF treatment but also by the 1- and 7-day SiNF treatments (Table 1). Many DEGs encoding WRKY proteins were induced by both the SiAF and the SiNF treatments (Table 1). The degree of induction by the 7-day SiAF treatment was greater than that by the 1-day SiAF treatment or the 7-day SiNF treatment (Additional file 3: Table S2).

PR proteins are categorized according to their functions, and they are involved in defense responses to biotic and abiotic stresses (van Loon et al. [2006]). The expression of genes encoding PR proteins was influenced by both the SiAF and the SiNF treatments, but the direction of the gene response was dependent on the gene family (Table 1). The number of DEGs and their degree of induction were greater after the 7-day SiAF treatment than the 7-day SiNF treatment (Table 1).

### Others

**Figure 4:**
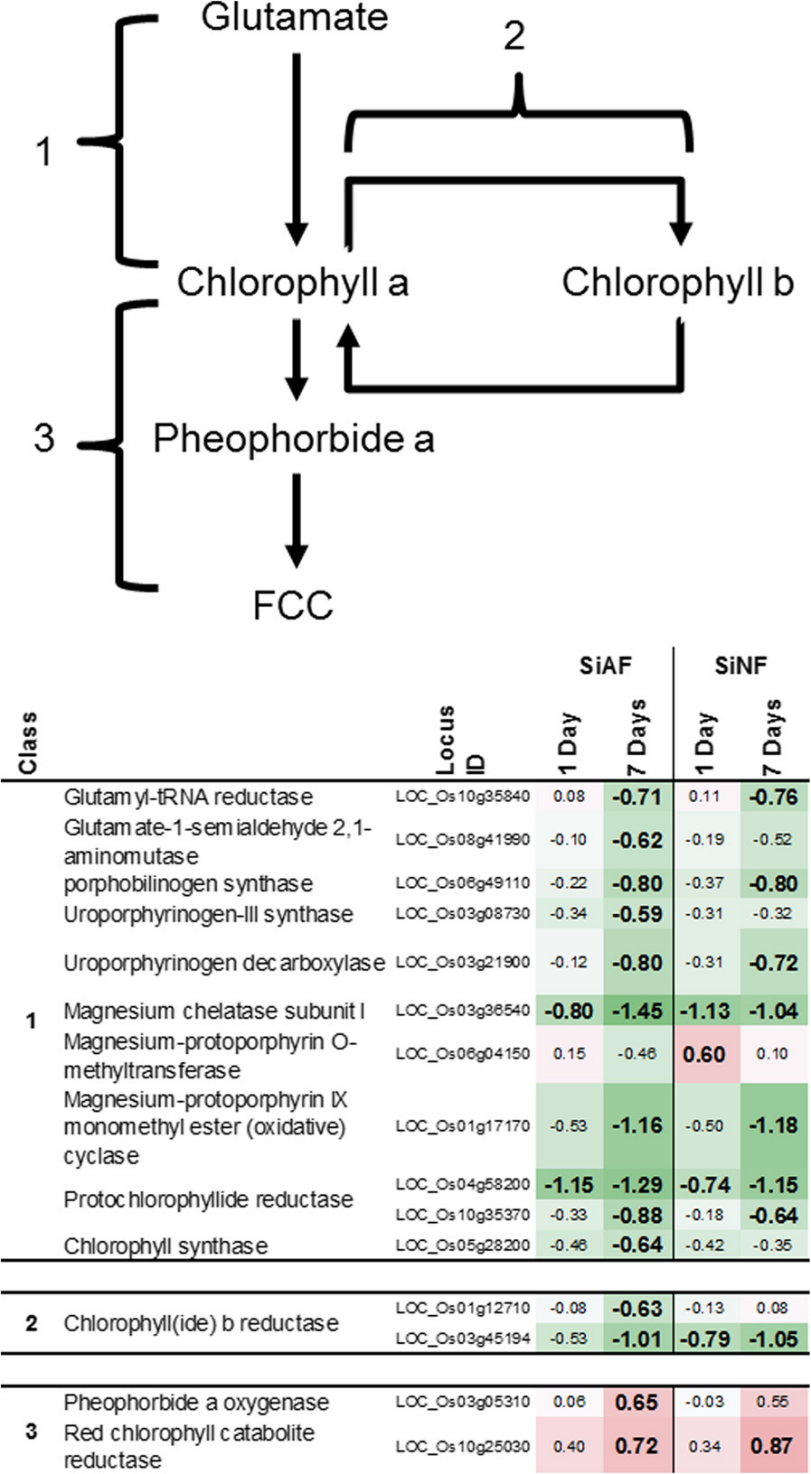
**Chlorophyll synthesis and degradation pathway.** 1: chlorophyll a synthesis, 2: conversion between chlorophyll a and b, 3: degradation of chlorophyll a. FCC: Fluorescent chlorophyll catabolite. This pathway is referenced in the KEGG database (Kanehisa et al. [2012]).

Genes involved in chlorophyll metabolism responded to the 7-day SiAF and the SiNF treatments (Figure 4). Moreover, the direction of the gene response was dependent on the process: the genes for chlorophyll synthesis and the conversion between chlorophyll a and b were suppressed by the 7-day SiAF and the SiNF treatments; however, the genes for chlorophyll degradation, such as pheophorbide a oxygenase (LOC_Os03g05310), were activated by these treatments (Figure 4). The number of DEGs was greater after SiAF treatment than after SiNF treatment (Figure 4).

Genes encoding cell wall components also responded to fog treatment, and the direction of the gene response to fog treatment was also dependent on the gene function (Table 1). The genes encoding expansin were suppressed by the SiAF and the SiNF treatments. The suppression occurred in both the 1- and 7-day SiAF treatments, but it only occurred in the 7-day SiNF treatment (Table 1). On the other hand, the genes encoding cellulose synthase and synthase-like protein were induced by both the SiAF and the SiNF treatments; the induction was also detected in both the 1- and 7-day treatments (Table 1).

### Comparison of the expression profiles between rice plants treated with various stresses

**Figure 5:**
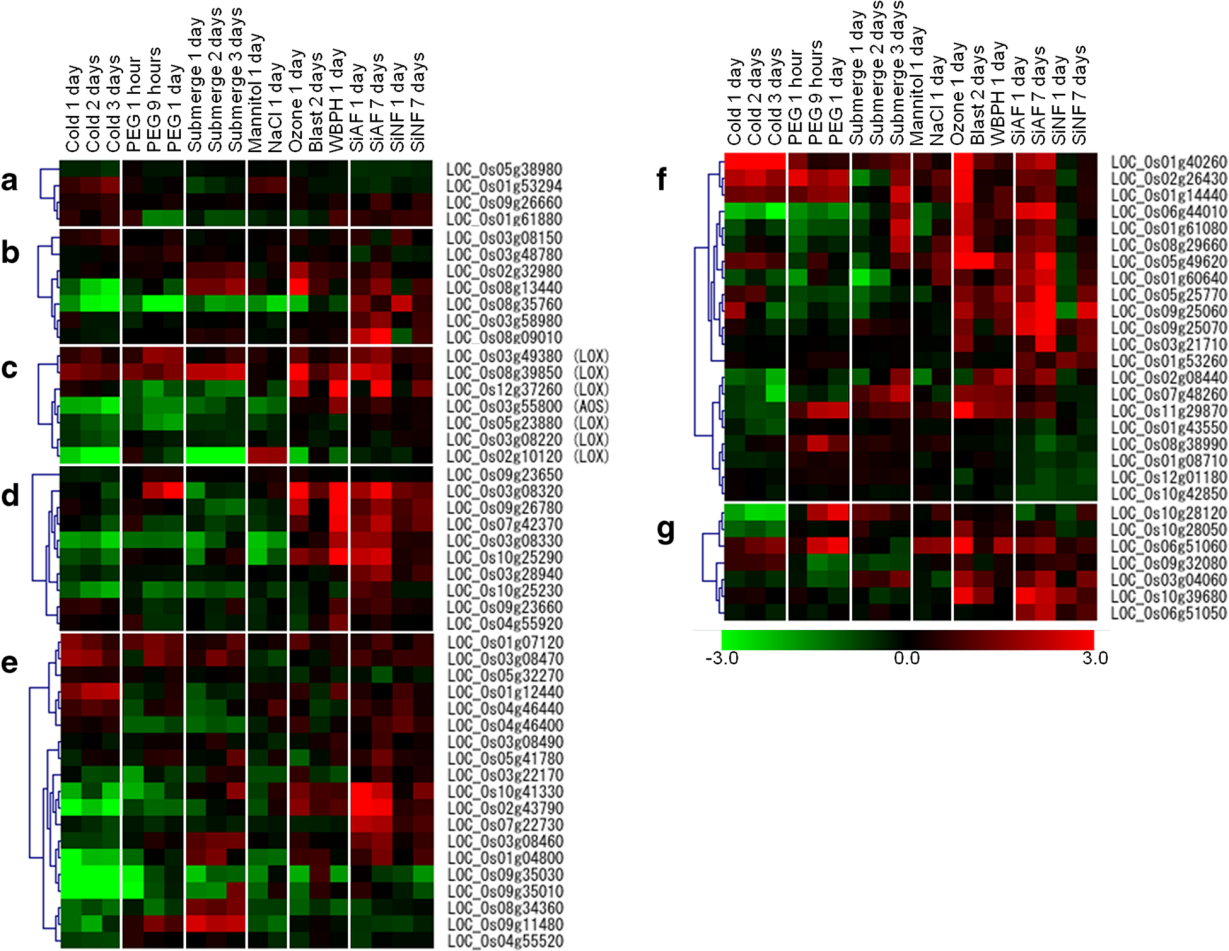
**Comparison of gene responses to various stress factors in rice.** Cold: 10°C; PEG: 25% polyethylene glycol 600; Mannitol: 260 mM mannitol; NaCl: 150 mM NaCl; Ozone: 0.2 ppm ozone; Blast: infection with *Magnaporthe grisea*; WBPH: infestation with whitebacked planthopper. The GEO IDs for these transcriptome datasets are GSE2415, 7532, 8811, 9450, and 11157. Red and green colors indicate up- and down-regulation of gene expression, respectively. Numbers represent the log^2^ ratio (signal intensity of the treated sample/signal intensity of the control sample). **a)**: NADPH oxidase, **b)**: germin, **c)** lipoxygenase(LOX) and allene oxide synthase (AOS), **d)**: JAZ(TIFY), **e)**: AP2/EREBP, **f)**: WRKY, and **g)** chitinase. The gene list of each category is based on KEGG ontology (Kanehisa et al. [2012]), Plant transcription factor database (Zhang et al. [2011]) and MSU OSA1 (Ouyang et al. [2007]), and the gene list in **e)**-**g)** is only fog-treatment responded gene. Original data are in Additional file 3: Table S2.

To characterize the gene responses to fog treatment, we compared the expression profiles between rice plants treated with either SiAF or SiNF and those exposed to various other stress treatments (Figure 5, Additional file 3: Table S2).

The expression of genes encoding NADPH oxidase was induced by both fog treatments and some other stress treatments (Figure 5); the gene response was dependent on the stress conditions. Two NADPH oxidase genes (LOC_Os01g61880 and LOC_Os09g26660) were induced by the SiNF, SiAF, whitebacked planthopper WBPH inoculation, and chilling treatments but were suppressed by the PEG, submergence, and ozone treatments (Figure 5a). One gene (LOC_Os01g53294) was induced by the chilling, mannitol, and salt treatments but not by the SiAF, SiNF, or biotic stresses (Figure 5a). Some germin genes were induced by the SiAF, SiNF, biotic, and some abiotic stresses but were suppressed by the chilling and PEG treatments (Figure 5b).

The expression of genes that function in organelles did not change in plants treated with either SiAF or SiNF (Table 1) and also showed no response in blast-infected plants (Additional file 3: Table S2). However, the expression of many such genes changed in plants subjected to abiotic stresses (Additional file 3: Table S2). Many genes encoding glutaredoxin responded to the abiotic stresses but not to the SiAF, SiNF, or biotic stresses (Additional file 3: Table S2). Many GST genes were induced by the abiotic stresses as well as the SiAF and SiNF treatments, although the specific genes that responded to the abiotic stresses differed from those that responded to the fog treatments and the biotic stresses (Additional file 3: Table S2).

The expression of genes involved in JA synthesis varied under both the biotic and abiotic stresses (Figure 5c). The genes encoding LOX and allene oxide synthase (AOS), both of which function in chloroplasts [25], were induced by the biotic stresses and the SiAF and SiNF treatments, although some of these genes were suppressed by the abiotic stresses (Figure 5c). The expression of the JAZ genes was activated by the biotic stresses and the ozone, SiAF, and SiNF treatments and suppressed by other abiotic stresses (Figure 5d). Many types of stress factors induced genes encoding AP2-EREBP and WRKY proteins, although the specific responsive genes were also dependent on the type of stress (Figure 5e and f). Genes that were highly induced by the SiAF treatment were also induced by the biotic stresses but were suppressed by the abiotic stresses such as chilling and drought. *WRKY45* (LOC_Os05g25770) gene expression was induced by the biotic stresses and the ozone, SiAF, and SiNF treatments but was suppressed by the abiotic stresses (Figure 5f and Additional file 3: Table S2). The genes encoding PR proteins also responded to many types of stress factors, and these gene responses were also dependent on the type of stress (Additional file 3: Table S2). Among chitinase genes, the DEGs activated by the SiAF and the SiNF treatments were similar to those activated by the biotic stresses (Figure 5g).

## Discussion

Both the SiAF and the SiNF treatments affected gene expression in rice plants (Figure 1). The member of DEG in plants subjected to the 7-day SiAF treatment were similar to those in plants subjected to either the 1-day SiAF treatment or the 7-day SiNF treatment (Figure 1), indicating that continuous SiNF and SiAF treatments result in similar conditions for rice plants. The 14-day SiNF treatment was not sufficient to produce visual symptoms; however, rice plants treated with SiAF for 14 days demonstrated visual symptoms (Ito et al. [2011]). This difference in visual symptoms between SiAF and SiNF treatment correlates with the concentration of sulfate (SO_4_^2−^) or the pH.

Accumulated ROS may cause symptoms such as chlorosis. ROS generation has been reported in plants exposed to acid rain (Gabara et al. [2003]; Velikova et al. [2000]; Wyrwicka and Sklodowska [2006]), and SO_4_^2−^ has been shown to cause acid rain-induced disease symptoms (Lee et al. [2006]). The activation of NADPH oxidase and ROS accumulation in the apoplast have been reported in spinach plants treated with gaseous SO_2,_ which is a precursor of SO_4_^2−^ (Li et al. [2007]). Moreover, proton stress has been reported to cause ROS accumulation in the apoplast of wheat (Song et al. [2010]). ROS accumulation from NADPH oxidase is similar to the oxidative burst induced by wounding or pathogen infection in the apoplast (Gill and Tuteja [2010]; Miller et al., [2009]; Miller et al. [2010]; Torres [2010]; Zurbriggen et al. [2010]). In this study, both fog treatments activated the same genes for encoding NADPH oxidase induced by biotic stresses (Figure 5). Moreover, stimulus such as wounding and pathogen infection also activates JA synthesis (Fujita et al. [2006]; Pré et al. [2008]). Both of the fog treatments also induces the genes for JA synthesis. On the other hand, ROS synthesis is also induced by abiotic stresses, such as cold and drought. However, the expression profile of rice undergoing these abiotic stresses is different from rice exposed to the fog treatment (Figure 5). These findings indicate that ROS synthesis in the apoplast and JA synthesis are induced by both fog treatments and that these molecules may be used as a signal molecule to induce the defense system for responding to wounding and biotic stress.

Fog treatment induced not only the genes encoding NADPH oxidase for O_2_^−^ generation but also the genes encoding germins for the reduction of O_2_^−^ in the apoplast. Germin was also shown to produce H_2_O_2_ and CO_2_ from oxalate and H_2_O in the apoplast (Banerjee and Maiti [2010]; Carrillo et al. [2009]; Gill and Tuteja [2010]; Manosalva et al. [2009]; Miller et al [2010]; van Loon et al. [2006]). These genes were induced more in plants treated with SiAF than in those treated with SiNF, indicating that more H_2_O_2_ had accumulated in plants treated with SiAF than in plants treated with SiNF. Moreover, of the five germin genes induced by the fog treatment, four genes were located on Chromosome 8, and two genes (LOC_Os08g09010, and 09060) were members of OsGER4. The germin genes located on Chromosome 8 are related to pathogen resistance, with the OsGER4 family contributing the most to the resistance (Manosalva et al. [2009]). The expression of the germin genes located on Chromosome 8 was induced by the fog treatment and biotic stresses but was not induced by chilling or osmotic stresses (Figure 5). This result also indicates that the fog treatments cause stress for rice plants and that the responses to both fog treatments were similar to the responses to biotic stress.

It is known that the accumulation of ROS causes the induction of disease symptoms (Tenhaken et al. [1995]); however, rice plants with 7 days of the SiAF treatment did not show disease symptoms. Symptoms were detected after as early as a few days of acid rain treatment in *Arabidopsis,* tomato, and cucumber plants (Gabara et al. [2003]; Lee et al. [2006]; Wyrwicka and Sklodowska [2006]), suggesting that rice plants are relatively tolerant to acid fog, which is consistent with a previous report (Wang et al. [2008]). These data also imply that rice has other antioxidant systems. The difference between rice and susceptible plants such as *Arabidopsis* is apparent in the concentration of endogenous SA. The rice plant contains much higher levels of SA than *Arabidopsis* (>50 times higher) (Yang et al. [2004]) therefore, rice is an SA-insensitive plant. The SA-deficient rice plant is an SA-sensitive plant and showed hypersensitivity to oxidative damage, as indicated by a high level of ROS accumulation. These results suggest that SA is an antioxidant compound (Yang et al. [2004]). Moreover, the level of SA in rice plants may determine the degree of acid fog resistance.

## Conclusions

This study suggests that the SiAF treatment causes stress resulting in ROS generation in the apoplast and that it induces defense responses similar to those from biotic stresses, even though an acid fog treatment is an abiotic stress for rice plants. Moreover, the gene responses to the long-term SiNF treatment were similar to those to the SiAF treatment, indicating that fog treatments are a stress to rice plants. At present, the mechanism for ROS and JA formation from a fog treatment remains unknown and should be addressed in future research.

## Methods

### Growth and fog treatments of plants

Seeds of *Oryza sativa* L., cv. Hinohikari rice were germinated on moist soil (Engeibaido, Kureha Corp., Japan) and grown at 25°C with 70% relative humidity under natural light in a greenhouse. After 7 days, the seedlings were transferred to porous pots (5-cm diameter, 6-cm height) containing gravel (Hydroball, Toshi-Engei Co., Ltd., Japan), dipped in aerated liquid medium (1/1000-diluted Hyponex with Hoagland No. 2 micronutrients and Fe-EDTA), and cultured for another 7 days in the greenhouse. These potted seedlings were then transferred to fog cabinets placed in a growth chamber and exposed to simulated neutral (pH 5.6: SiNF) or acid (pH 3.0: SiAF) fog for 6 h (9:00-15:00) each day for 7 days (Ito et al. [2011]). During the 7-day treatment, the seedlings were kept under a 14-h (5:00-19:00) light/10-h dark cycle, and the light intensity during the light period was 400-500 μmol m^−2^ s^−1^ at the bottom of the fog cabinet. Other conditions inside the growth chamber included temperature at 25/20°C and 55/75% relative humidity during the light/dark periods. Deionized water was used as the source of SiNF, and the source of SiAF was prepared by adding sulfuric acid to deionized water. The fog was evolved from these sources with an ultrasonic humidifier (FT-10, UCAN Co., Ltd.) and introduced into the fog cabinet through fan-induced air flow. As a control, rice seedlings were treated similarly but in the absence of fog treatment.

### RNA extraction

Total RNA samples were extracted from leaves pooled from 5 independent plants from the same experiment using the RNeasy Plant Mini kit (Qiagen). For the microarray experiment, we prepared 18 RNA samples (3 conditions (control, SiAF, and SiNF) × 2 time points (1 and 7 days after the beginning of fog treatment) × 3 biological replicates). The concentration and quality of total RNA were examined using a spectrophotometer (Nanodrop ND-1000, Nanodrop Technologies) and a BioAnalyzer (G2938A, Agilent Technologies), respectively.

### Microarray analyses

We performed microarray analyses using the 2-dye method, which directly compares expression profiles between 2 samples on the same microarray slide. The microarray experiment and data analysis methodology have been previously described in detail (Satoh et al. [2010]). In brief, cyanine 3(Cy3)- or cyanine 5 (Cy5)-labeled complementary RNA (cRNA) samples were synthesized from 850 ng total RNA using a Low Input RNA labeling kit (Agilent Technologies). In this study, samples treated with SiAF and SiNF were labeled by Cy3, and those without fog treatment were done by Cy5 (Table 2). Hybridization solution was prepared with 825 ng each of Cy3- and Cy5-labeled cRNA (Table 2) preparations using the In situ Hybridization Plus kit (Agilent Technologies). Hybridization and washing of microarray slides (GPL7252, 43494 probes [Edgar et al. [2002]) were performed following manufacturer’s protocols. After washing, the slide image files were generated by a DNA microarray scanner (G2505B; Agilent Technologies). Cy3 and Cy5 signal intensities were extracted from the image files and normalized in each array using the Feature Extraction software version 9.5 (Agilent Technologies). Signal intensities among all microarray data were normalized according to the quantile method (global normalization) using the EXPANDER software version 4.1 (Ulitsky et al. [2010]). A gene was considered to be expressed if the average signal intensity of the gene was higher than the median global signal intensity in least at one condition; otherwise, the gene was not considered to be expressed. A DEG was defined as an expressed gene for which 1) the difference of signal intensity between treatment and control samples was greater than 1.5-fold and 2) the difference in gene expression between the 2 samples was significant at P-value ≤ 0.05 by a paired t test (permutation: all; FDR collection: adjusted Bonferroni method). Data processing was performed using the Mev software version 4.3 (Saeed et al. [2006]). The results of the microarray analysis used in this study (series number: GSE31196) are available at the NCBI-GEO (Edgar et al. 2003).

**Table 2:** Sample combinations for microarray analysis

**Experiment**	**Length of treatment**	**Cy3**	**Cy5**	**GEO ID**^**1**^
1	1 day	SiAF	Control	GSM773394
2	1 day	SiAF	Control	GSM773395
3	1 day	SiAF	Control	GSM773396
4	7 day	SiAF	Control	GSM773397
5	7 day	SiAF	Control	GSM773398
6	7 day	SiAF	Control	GSM773399
7	1 day	SiNF	Control	GSM773400
8	1 day	SiNF	Control	GSM773401
9	1 day	SiNF	Control	GSM773402
10	7 day	SiNF	Control	GSM773403
11	7 day	SiNF	Control	GSM773404
12	7 day	SiNF	Control	GSM773405

^1^ID of the NCBI GEO database (Edgar et al. 2003).

### Comparative analysis of the transcriptome data

The transcriptome data of rice plants subjected to abiotic stress treatment were derived from the GSE2415, 7532, and 11157 datasets deposited on NCBI GEO (Edgar et al. 2003). From these data, we selected 12 microarray datasets corresponding to rice plants treated with chilling (10°C, 3 time points), 25% polyethylene glycol (PEG) 600 (simulated drought stress, 3 time points), 260 mM mannitol (stimulated osmotic stress, one time point), 150 mM NaCl (one time point), submergence (3 time points), and 0.2 ppm ozone (one time point). The transcriptome data of rice plants with biotic stress treatment were derived from GSE9450 (infection with blast fungus for 2 days) and GSE8811 (infestation with whitebacked planthopper for 2 days). Comparisons could be made between our data and other stress data for 13,386 of 22,328 expressed genes in our study and for 2,957 of 13,401 genes that responded to the SiAF and/or the SiNF treatments.

### RT-PCR

cDNA fragments for rice plant transcripts were synthesized from 1,000 ng total RNA with 50 pmol of random hexamer using the SuperScript III reverse transcriptase (Invitrogen). The resultant reaction mixtures containing cDNA fragments were serially diluted 4 times and then used for PCR. To examine the expression levels of the rice genes, PCR was performed with 4 μl of a diluted cDNA reaction mixture in a final volume of 20 μl using Taq DNA polymerase (New England Biolabs). The gene-specific primers designed by Primer 3 (Rozen and Skaletsky [2000]) and used for amplification of the selected rice gene transcripts are listed in Additional file 2: Figure S1. Primers for the rice genes were designed to generate PCR products of 150 − 720 bp in length. A ribosomal protein gene (LOC_Os03g34040), whose expression levels remained nearly constant under all experimental conditions, was used as a control for the gene expression analysis by RT-PCR. The cycling program consisted of an initial denaturation for 1 min at 95°C, followed by 30 to 40 cycles of denaturation at 95°C for 15 s, annealing for 15 s, and extension at 68°C for 45 s, with a final extension of 1 min at 68°C. The annealing temperatures are indicated in Additional file 2: Figure S1.

## Competing interests

The authors declare that they have no competing interests.

## Authors’ contributions

KS performed the microarray experiment, RT-PCR and wrote the manuscript. SS performed the sampling and RNA extraction from rice leaves. SI and HideS performed the cultivation and fog treatments of rice plants. HikaS and SK conceived and designed the study and critically reviewed the manuscript. All authors read and approved the final manuscript.

## Additional files

## Supplementary Material

Additional file 1: Table S1. A list of genes expressed in fog-treated plants. Bold numbers indicate differentially expressed genes in plants subjected to each treatment. ^1^based on MSU OSA1 version 5 (Ouyang et al. [2007]). Click here for file

Additional file 2: Figure S1. Differentially expressed genes evaluated by RT-PCR. The numbers are log2-based differential expression ratios from the microarray analysis. ns: log^2^-based differential expression ratio of the gene not significantly differentially expressed. A ribosomal protein gene (LOC_Os03g34040) whose expression levels remained nearly constant under all experimental conditions was used as a control for gene expression analysis by RT-PCR. Click here for file

Additional file 3: Table S2. Comparison of DEGs among fog- and other treated plants. A) The number of differentially expressed genes in each category. Each column shows the number of induced genes/number of suppressed genes in each category.^1^ B) A list of the genes in each category. Bold numbers indicate differentially expressed genes in plants subjected to each treatment. Click here for file
