# Supplementary figures and images for: Gene response in rice plants treated with continuous fog influenced by pH, was similar to that treated with biotic stress
Source: Rice (N Y). 2014 Jun 11;7(1):10. doi: 10.1186/s12284-014-0010-9 (PMC4077630; doi:10.1186/s12284-014-0010-9)

## Slide 1
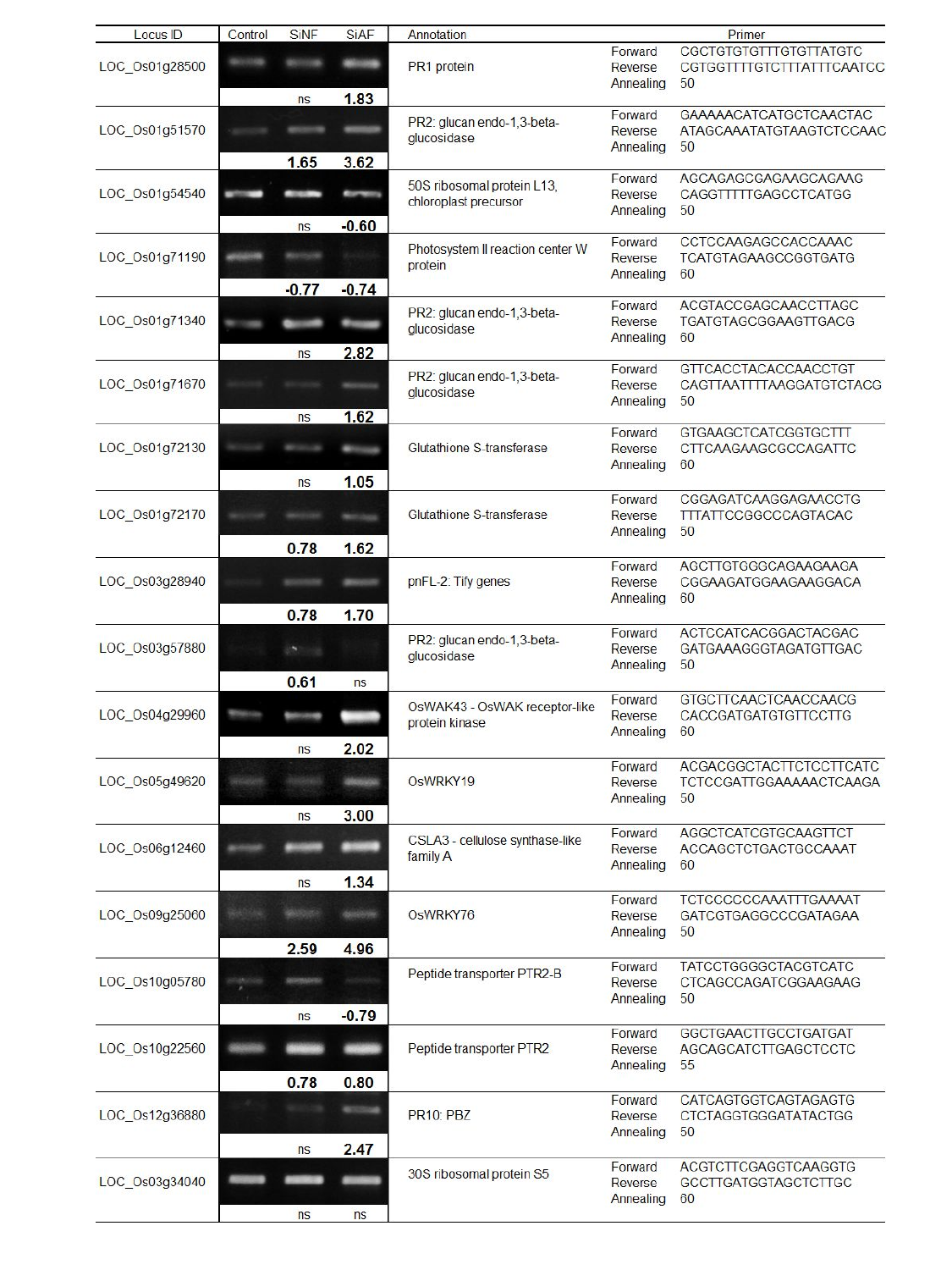

Supplement: Additional file 2: Figure S1. — Differentially expressed genes evaluated by RT-PCR. The numbers are log2-based differential expression ratios from the microarray analysis. ns: log2-based differential expression ratio of the gene not significantly differentially expressed. A ribosomal protein gene (LOC_Os03g34040) whose expression levels remained nearly constant under all experimental conditions was used as a control for gene expression analysis by RT-PCR. [file s12284-014-0010-9-S2.ppt]
